# Investigation of *Hyalomma turanicum* and *Hyalomma asiaticum* in Pakistan, with notes on the detection of tickborne Rickettsiales

**DOI:** 10.3389/fvets.2024.1500930

**Published:** 2024-12-23

**Authors:** Zafar Ullah, Iram Liaqat, Mehran Khan, Abdulaziz Alouffi, Mashal M. Almutairi, Dmitry A. Apanaskevich, Tetsuya Tanaka, Abid Ali

**Affiliations:** ^1^Microbiology Lab, Department of Zoology, Government College University, Lahore, Punjab, Pakistan; ^2^Department of Zoology, Abdul Wali Khan University Mardan, Mardan, Khyber Pakhtunkhwa, Pakistan; ^3^King Abdulaziz City for Science and Technology, Riyadh, Saudi Arabia; ^4^Department of Pharmacology and Toxicology, College of Pharmacy, King Saud University, Riyadh, Saudi Arabia; ^5^United States National Tick Collection, The James H. Oliver Jr. Institute for Coastal Plain Science, Georgia Southern University, Statesboro, GA, United States; ^6^Department of Biology, Georgia Southern University, Statesboro, GA, United States; ^7^Laboratory of Animal Microbiology, Graduate School of Agricultural Science/Faculty of Agriculture: Tohoku University, Aoba-ku, Sendai, Japan

**Keywords:** *Hyalomma turanicum*, *Hyalomma asiaticum*, *Rickettsia aeschlimannii*, *Anaplasma* sp., *Ehrlichia* sp., coinfection, phylogeny

## Abstract

There is limited information on the occurrence of *Hyalomma turanicum* and *Hyalomma asiaticum* ticks, as well as associated *Rickettsia, Anaplasma,* and *Ehrlichia* species in Pakistan. Addressing this knowledge gap, the current study aimed at morphomolecular confirmation of these ticks and molecular assessment of associated Rickettsiales bacteria (*Rickettsia, Anaplasma*, and *Ehrlichia* spp.) in Balochistan, Pakistan. A total of 314 ticks were collected from 74 of 117 (63.2%) hosts, including 41 of 74 (55.4%) goats and 33 of 74 (44.5%) sheep. Subsequently, a subset of microscopically identified ticks was subjected to DNA extraction and PCR to amplify 16S rDNA and *cox1* fragments. Additionally, *gltA*, *ompA,* and *ompB* fragments were targeted for *Rickettsia* spp. and 16S rDNA fragments for both *Anaplasma* and *Ehrlichia* spp. The 16S rDNA and *cox1* sequences of *Hy. turanicum* demonstrated 100% identity with those of the same species previously reported from Pakistan. The 16S rDNA and *cox1* sequences of *Hy. asiaticum* exhibited 99.52 and 100% identities, respectively, with corresponding species reported from China, Kazakhstan, and Turkey. The *gltA, ompA,* and *ompB* fragments associated with *Hy*. *turanicum* showed 100% identities with *Rickettsia aeschlimannii* reported from Egypt, Italy, Kazakhstan, Kenya, Pakistan, and Senegal. The 16S rDNA sequences of *Anaplasma* sp. and *Ehrlichia* sp. associated with both *Hy*. *asiaticum* and *Hy*. *turanicum* exhibited 99.67 and 100% identities with unknown *Anaplasma* sp. and *Ehrlichia* sp. reported from Morocco and Pakistan, respectively. In the 16S rDNA and *cox1* phylogenetic trees of ticks, *Hy*. *turanicum* and *Hy*. *asiaticum* from the current study clustered with their respective species. Similarly, in *gltA, ompA,* and *ompB* phylogenetic trees of *Rickettsia*, *R. aeschlimannii* of the present study clustered with the same species, whereas *Anaplasma* sp. and *Ehrlichia* sp. of this study clustered with undetermined *Anaplasma* spp. and *Ehrlichia* spp. in the 16S rDNA phylogenetic tree of Anaplasmataceae. Among the DNA samples from the screened ticks, a coinfection rate of *R. aeschlimannii*, *Anaplasma* sp., and *Ehrlichia* sp. (2 of 80, 2.5%) was observed in *Hy. turanicum*, whereas individual infection rates were noted as follows: *R. aeschlimannii* (8 of 80, 10%), *Anaplasma* sp. (5 of 80, 6.3%), and *Ehrlichia* sp. (5 of 80, 6.3%). This study marks the first record of molecular characterization of *Hy*. *turanicum* and *Hy*. *asiaticum* as well as the detection of associated *R. aeschlimannii*, *Anaplasma* sp., and *Ehrlichia* sp. in Balochistan, Pakistan.

## 1 Introduction

Ticks are hematophagous ectoparasites and leading vectors of important tickborne pathogens that affect all classes of terrestrial and semi-terrestrial animals, including livestock. Losses due to tick infestation could be either direct or indirect and are affected by certain climatic factors, including fluctuation in temperature, humidity, photoperiod, and vegetation, as well as host availability (1–3). The *Hyalomma* genus is among the prominent genus of ticks found in Asia, Africa, and Southern Europe with 27 reported species (4), while in Pakistan, 14 species of this genus have been reported (5–7). The several species complexes in this genus share many morphological traits, making accurate identification challenging. Within *Hyalomma* (*Euhyalomma*) *marginatum* complex, *Hyalomma glabrum* Delpy 1949, *Hyalomma isaaci* Sharif 1928, *Hy. marginatum* Koch 1844, *Hyalomma rufipes* Koch 1844, and *Hyalomma turanicum* Pomerantzev 1946 are closely related species (8). Of these species, *Hy*. *turanicum* is found in Africa and Asia (Afghanistan, Bahrain, China, Egypt, India, Iran, Iraq, Israel, Jordan, Kazakhstan, Kyrgyzstan, Nepal, Oman, Pakistan, Qatar, Saudi Arabia, Syria, Tajikistan, Turkmenistan, Uzbekistan, and Yemen) (9, 10). Within *Hyalomma* (*Euhyalomma*) *asiaticum* complex, *Hyalomma asiaticum* Schulze & Schlottke 1929, *Hyalomma dromedarii* Koch 1844, *Hyalomma impeltatum Schulze* & Schlottke 1929, *Hyalomma schulzei* Olenev 1931, and *Hyalomma somalicum* Tonelli Rondelli 1935 are closely related (8, 11). Among these species, *Hy*. *asiaticum* is limited to Asia (Afghanistan, Armenia, Azerbaijan, China, Georgia, Iran, Iraq, Kazakhstan, Kyrgyzstan, Mongolia, Pakistan, Russia, Syria, Tajikistan, Turkey, Turkmenistan, and Uzbekistan) (9, 10). Key morphological features of these species are adanal plates, spiracular plates, setae, genital aperture, and scutum/conscutum (12, 13).

Morphological-based identification of *Hyalomma* species creates more challenges, which resulted in the re-description of its several species (14, 15). Recent studies of molecular identification have improved our understanding of the inter- and intra-specific diversity of *Hyalomma* ticks, which may help in understanding its taxonomy and epidemiology (9, 16, 17).

Within the order Rickettsiales, the genus *Rickettsia* consists of obligate intracellular *Gram*-negative bacteria that affect veterinary and human health by causing various diseases such as Rocky Mountain spotted fever (18, 19). In this order, *Ehrlichia* species have unique cell tropisms within the vertebrate hosts and are causative agents of tickborne fever in ruminants, while *Anaplasma* species cause human granulocytic anaplasmosis (20, 21). Several *Rickettsia*, *Anaplasma*, and *Ehrlichia* species of medical importance are vectored by *Hyalomma* ticks, including *Hy. turanicum* and *Hy. asiaticum* (22, 23). Moreover, these tick species are also competent vectors of various viruses, including the Crimean–Congo hemorrhagic fever (CCHF) viruses (24). In Pakistan, *Hyalomma* ticks are expected to be associated with tickborne diseases such as babesiosis, theileriosis, rickettsiosis, and CCHF (7, 25–27). Pakistan’s economy is mostly based on agriculture, which contributes ≈ 24% to its gross domestic product and employs ≈ 40% of its labor force (53). Consequently, these TBDs can cause significant damage to the country’s economy.

Though *Hyalomma* ticks are highly important in both veterinary and medical aspects, morphomolecular studies of *Hy*. *turanicum* and *Hy*. *asiaticum* in Pakistan in general and in Balochistan province in particular are limited. Additionally, *Hy*. *turanicum* and *Hy*. *asiaticum* ticks from Pakistan have been largely overlooked in terms of pathogen research, including *Rickettsia*, *Anaplasma,* and *Ehrlichia* spp. Therefore, the study represents the first attempt to investigate the occurrence of *Hy*. *turanicum* and *Hy*. *asiaticum* ticks and Rickettsiales bacteria associated with these tick species. This study specifically aims to determine whether *Hy*. *turanicum* and *Hy*. *asiaticum* are associated with *Rickettsia*, *Anaplasma*, and *Ehrlichia* spp. in the study region.

## 2 Materials and methods

### 2.1 Ethical consideration

Prior to performing this study, approval was given by the Advanced Studies and Research Board (REG-ACAD-ASRB-55/23/035) of the Government College University, Lahore, Pakistan. All animal or herd owners were verbally informed, and permission was taken from them.

### 2.2 Study area

**Figure 1:**
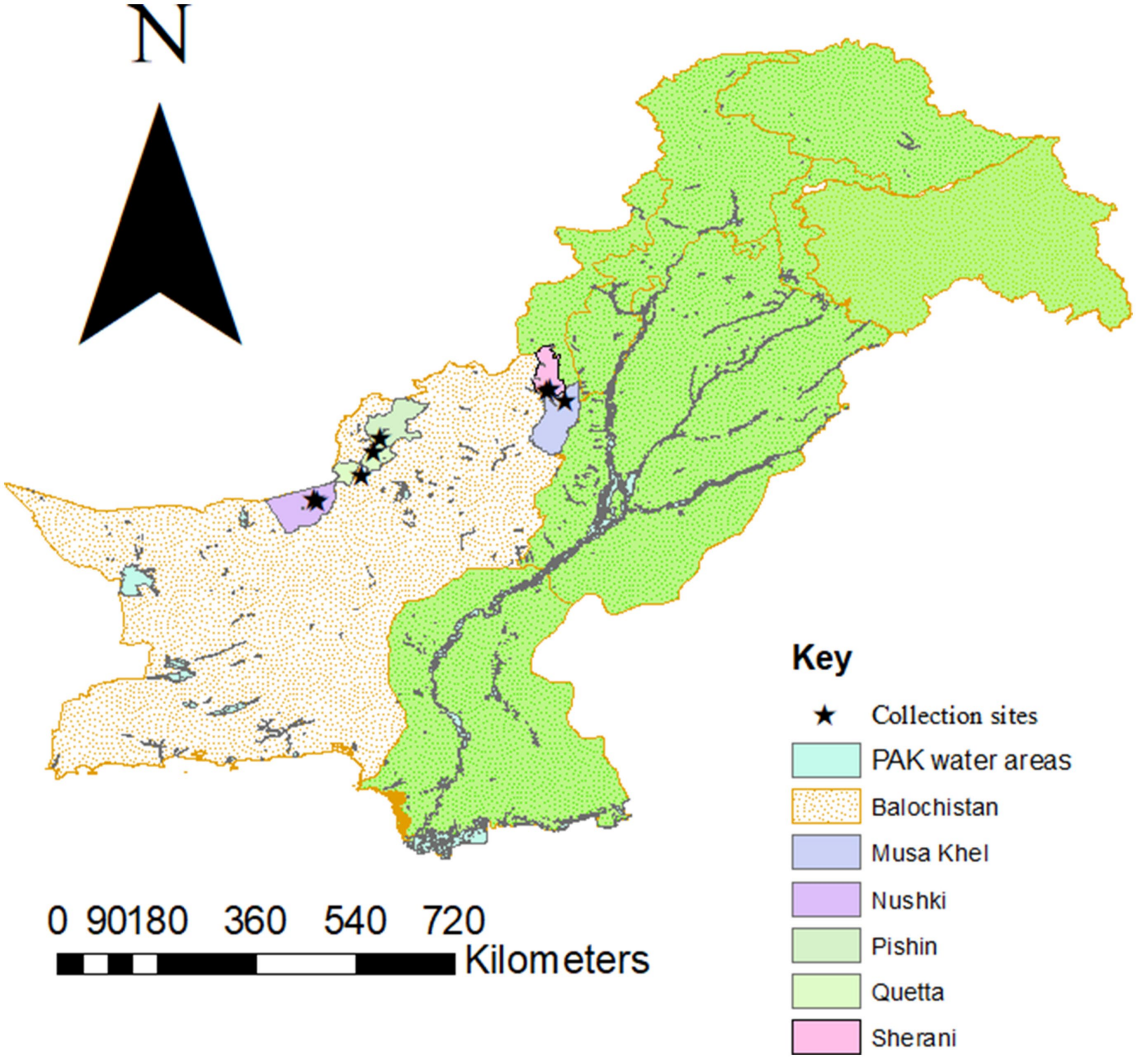
Map summarizing the study area.

The present study was executed in five districts of the Balochistan province, Pakistan: Musakhail (30°50′52.1″N 69°57′18.0″E), Nushki (29°28′27.1″N 65°39′57.2″E), Pishin (30°44′17.9″N 67°17′05.3″E), Quetta (30°10′47.6″N 67°05′38.8″E), and Sherani (29°46′60″ N 68°31′0″ E). For creating a map (Figure 1) using ArcGIS v. 10.3.1, the coordinates of the above-collected sites were documented using the Global Positioning System. The choice of these districts was strategic because they share borders with neighboring countries (Afghanistan and Iran), making them key areas for cross-border animal movement and trade. Additionally, Quetta, as the provisional capital, serves as a major hub for the transportation of livestock, further increasing the importance of these districts. Livestock, particularly sheep and goats, are more prevalent in these areas, forming the backbone of the local economy.

### 2.3 Collection and preservation of ticks

Ticks were collected from goats and sheep from April 2023 to April 2024 in the specified districts of Balochistan. To minimize potential damage to the specimens, a meticulous approach was employed, delicately detaching ticks from the skin of hosts using forceps. The collected specimens underwent a thorough cleaning process with distilled water prior to washing with 70% absolute ethanol. For preservation and further analysis, the ticks were stored in a solution made of 95% ethanol, 4% distilled water, and 1% glycerol.

### 2.4 Morphological identification

Tick specimens were meticulously examined under a stereo-zoom microscope (Luxeo 6Z, LABOMED, USA). Morphological identification was carried out at both the life stage and species level, with the help of established morphological identification keys specified to *Hyalomma* species (12, 15).

### 2.5 DNA extraction and PCR

A total of 80 morphologically identified ticks were randomly selected for molecular analysis, including four males and four females of each species per host per district. Subsequently, the homogenized specimens were incubated until dry, involving dissection and grinding with sterile scissors. DNA extraction followed the standard phenol–chloroform method with minor adjustments (28). After extraction, DNA was quantified using a NanoDrop spectrophotometer (NanoQ, Optizen, Daejeon, South Korea) and stored at −20°C for further analysis.

Conventional PCRs (GE-96G, BIOER, Hangzhou, China) were performed to amplify partial fragments of 16S ribosomal DNA and *cox1* genes of ticks from the extracted DNA (Table 1). Additionally, the extracted genomic DNA was molecularly assessed for rickettsial citrate synthase (*gltA*), outer membrane protein A and B (*ompa* and *ompB*), and Anaplasmataceae 16S rDNA (Table 1).

**Table 1:** List of primers and PCR conditions that were used for the molecular identification of ticks and pathogens.

Organisms	Genes	Primer name: Primers Sequences (5′–3′)	Amplicons size	PCR conditions	References
Ticks	16S rDNA	16S + 1: CCGGTCTGAACTCAGATCAAGT	460 bp	94°C—2 min, 34× (94°C—30 s, 55°C—30 s, 72°C—45 s), 72°C—7 min	(49)
		16S-1: GCTCAATGATTTTTTAAATTGCTG			
	*cox1*	HCO2198: TAAAC TTCAGGGTG ACCAAAAAATCA	710 bp	95°C—5 min, 40× (94°C—40 s, 48°C—60 s, 72°C—1 min), 72°C—10 min	(50)
		LCO1490: GGTCAACAAATCATAAAGATATTGG			
*Rickettsia*	*gltA*	CS-78: GCAAGTATCGGTGTGAGGATGTAAT	401 bp	95°C—3 min, 40 × (95°C—15 s, 56°C—30 s, 72°C—30 s) 72°C—7 min	(42)
		CS-232: GCTTCCTTAAAATTCAATAAATCAGGAT			
	*ompA*	Rr190.70: ATGGCGAATATTTCTCCAAAA	532 bp	95°C—3 min, 35 × (95°C—20 s, 55°C—30 s, 60°C—2 min) 72°C—7 min	(51)
		Rr190.701: AGTGCAGCATTCGCTCCCCCT			
	*ompB*	120-M59: CCGCAGGGTTGGTAACTGC	862 bp	95°C—3 min, 40 × (95°C—30 s, 50°C—30 s, 68°C—1 min 30 s), 68°C–7 min	(41)
		120–807: CCTTTTAGATTACCGCCTAA			
*Anaplasma*/*Ehrlichia* spp.	16S rDNA	EHR16SD: GGTACCYACAGAAGAAGTCC	344 bp	95°C—3 min, 35× (95°C—30 s, 55°C—30 s, 72°C—1 min), 72°C—7 min	(52)
		EHR16SR: TAGCACTCATCGTTTACAGC			

Each PCR was performed in a 20 μL volume, including 1 μL forward and reverse primers each (concentration of 10 μM), 4 μL of “nuclease-free” PCR water, 2 μL of genomic DNA (100 ng/μL), and 12 μL of Dream*Taq* MasterMix (2X) (Thermo Fisher Scientific, Inc., Waltham, MA, USA). The primers and thermocycling conditions used in this study are listed in Table 1. To ensure the reliability of the PCRs, positive control samples containing DNA of *Hyalomma anatolicum* (for ticks) and DNA of *Rickettsia massiliae* (for bacterial species), and a negative control sample consisting of PCR water (nuclease-free), were part of each PCR. The amplified products of the tick and its pathogen from the PCRs were run on a gel made of 2% agarose. After PCR, the obtained bands were made visible under through UV from a Gel Documentation system (BioDoc-It™ Imaging Systems, UVP, LLC).

### 2.6 DNA sequencing and phylogenetic analyses

PCR-positive amplified products of the expected size were purified using the DNA Clean and Concentrator Kit (Zymo Research, Irvine, CA, USA) and submitted for bidirectional sequencing (Macrogen Inc., Seoul, South Korea) using the Sanger sequencing method. The resulting sequences were proceeded and purified through SeqMan v. 5 (DNASTAR, Inc., Madison, WI, USA) to remove low-quality and contaminated regions. Trimmed and consensus sequences were then analyzed using the Basic Local Alignment Search Tool (BLAST) at the National Center for Biotechnology Information (NCBI)^1^ (29). Sequences with high homology were downloaded in FASTA format. The downloaded sequences, along with an outgroup, were aligned using ClustalW (30) in the BioEdit alignment editor V.7.0.5 tool (Raleigh, NC, USA) (31).

Phylogenetic trees were constructed using MEGA-X (32) with the following specifications: Neighbor-Joining method, Tamura–Nei model, and 1,000 bootstrap replicates. Additionally, coding sequences were aligned using the MUSCLE alignment method (33).

## 3 Results

### 3.1 Morphological identification

**Figure 2:**
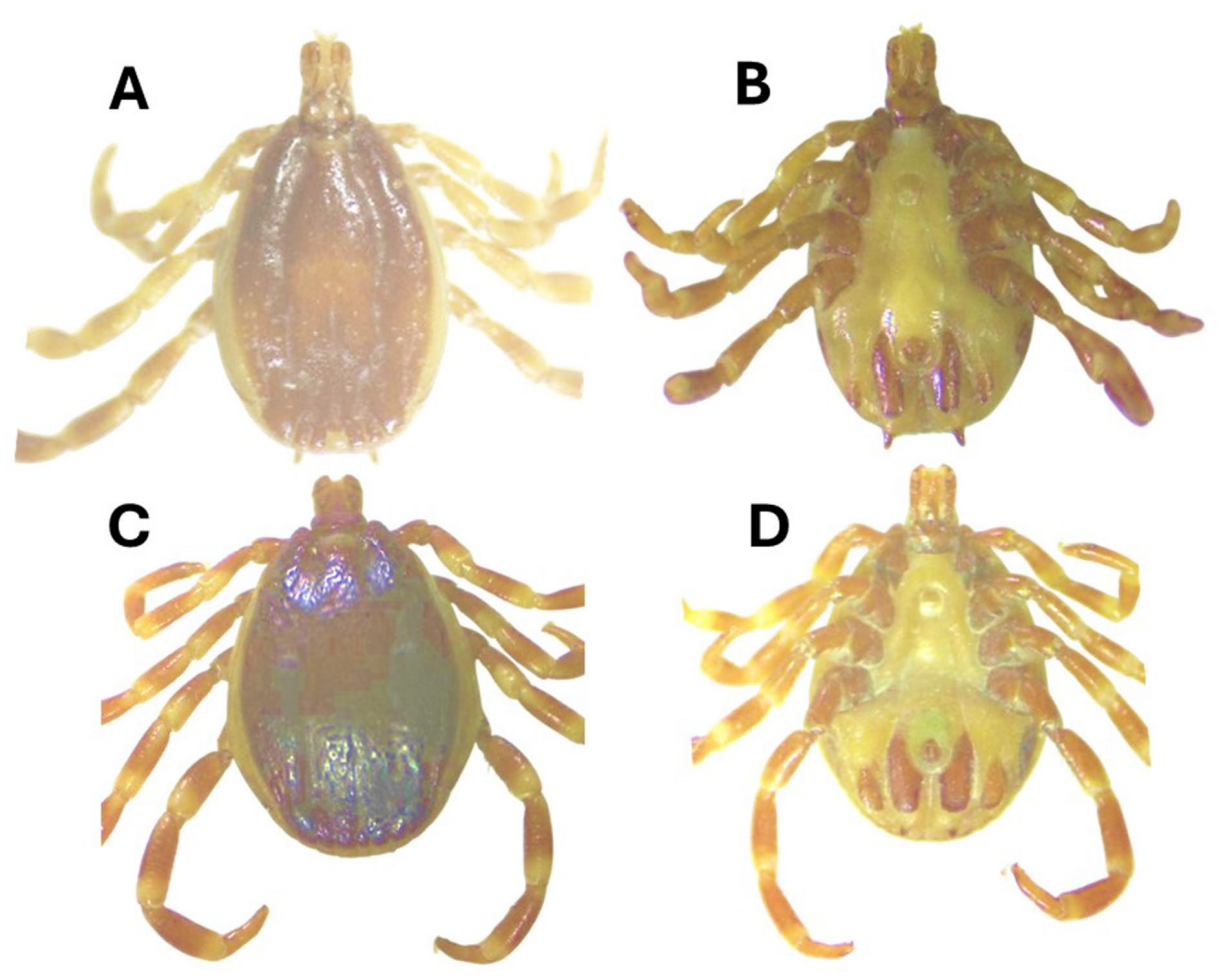
*Hyalomma asiaticum* male [**(A)** dorsal, **(B)** ventral] and *Hyalomma turanicum* male [**(C)** dorsal and **(D)** ventral] were collected in this study.

Morphologically, *Hy*. *turanicum* was identified based on different features. The conscutum exhibited a yellow–brown color, with large punctations being rare, while medium- and small-sized punctations were relatively dense and covered the entire conscutum. The perforated portion of dorsal prolongation of the spiracular plate is relatively narrow; circumspiracular setae moderately dense; proximally moderately sized dorsal ivory-colored enamel spot (Figures 2C, D). While the scutum of the female was dark, ranging from reddish brown to nearly black, the genital aperture was wide, deep, and rounded (U-shaped), with the vestibular portion of the vagina bulging to some extent.

The *Hy*. *asiaticum* tick was morphologically identified based on different features. The conscutum exhibited a yellow to red–brown coloration. The cervical grooves were remarkably deep, while the marginal grooves were short. The adanal plates were long, narrow, and nearly straight, tapering slightly posteriorly from the median projection. (Figures 2A, B). While the scutum of the female was yellow– to red–brown, the genital aperture was narrow, U-shaped with the vestibular portion of the vagina distinctly bulging.

### 3.2 Hosts and ticks

Altogether, 314 ticks were collected from five districts of Balochistan; Musakhail, Nushki, Pishin, Quetta, and Sherani. These ticks included 165 (56.18%) *Hy*. *asiaticum*, comprising 104 (53.07%) females and 61 (32.03%) males, as well as 149 (43.81%) *Hy*. *turanicum*, including 96 (54.77%) females and 53 (31.12%) males. All these ticks were collected from 74 of the 117 examined hosts, demonstrating an overall prevalence of infestation of 63.2%. Moreover, goats exhibited a high prevalence of infestation (41 of 74, 55.4%) compared to sheep (33 of 74, 44.5%). Detailed results about ticks, small ruminants, and Rickettsiales bacteria are summarized in Table 2.

**Table 2:** Collection sites of collected specimens and their molecular characterization with infection rate.

Collection sites	Hosts		Morphologically identified ticks				Tick subjected to PCR	PCR for pathogens							
	Name	Number of infested hosts/total		F	M	Total ticks		*R. aeschlimannii*				*Anaplasma* spp.		*Ehrlichia* spp.	
								*gltA*	*ompA*	*ompB*	Infection rate %	16S rDNA	Infection rate %	16S rDNA	Infection rate %
Musakhail	Goats, Sheep	9/11	*Hy*. *asiaticum*	23	14	37	4M, 4F	0	0	0	0	1M	1 (1.25)	1M	1 (1.25%)
	Goats, Sheep	6/7	*Hy*. *turanicum*	18	9	27	4M, 4F	1 M, 2F	1 M, 2F	1 M, 2F	3 (3.8)	2F	2 (2.5)	2F	2 (2.5%)
Nushki	Goats, Sheep	4/5	*Hy*. *asiaticum*	16	8	24	4M, 4F	0	0	0	0	0	0	0	0
	Goats, Sheep	6/8	*Hy*. *turanicum*	17	11	28	4M, 4F	1 M, 1F	1 M, 1F	1 M, 1F	2 (2.5)	0	0	0	0
Pishin	Goats, Sheep	12/13	*Hy*. *asiaticum*	18	8	26	4M, 4F	0	0	0	0	1F	1 (1.25)	1F	1 (1.25%)
	Goats, Sheep	8/11	*Hy*. *turanicum*	19	10	29	4M, 4F	0	0	0	0	0	0	0	0
Quetta	Goats, Sheep	13/17	*Hy*. *asiaticum*	26	17	43	4M, 4F	0	0	0	0	1F	1 (1.25)	1F	1 (1.25%)
	Goats, Sheep	11/16	*Hy*. *turanicum*	23	12	35	4M, 4F	0	0	0	0	0	0	0	0
Sherani	Goats, Sheep	9/11	*Hy*. *asiaticum*	21	14	35	4M, 4F	0	0	0	0	0	0	0	0
	Goats, Sheep	8/18	*Hy*. *turanicum*	19	11	30	4M, 4F	2 M, 1F	2 M, 1F	2 M, 1F	3 (3.8)	0	0	0	0
Total		74/117		200	114	314	80	8	8	8	8 (10)	5	5 (6.3)	5	5 (6.3)
Total infection rate (mean ± SD) (%)								20.1 (1.83 ± 2.99)				12.55 (1.14 ± 1.82)		12.55 (1.14 ± 1.82)	
Chi-square value (*p*-value)								2.97 (0.27)							

M, male; F, female.

### 3.3 Molecular outcomes

DNA was extracted from 80 ticks, including 40 *Hy. turanicum* and 40 *Hy. asiaticum*. Altogether, 53 bidirectional sequences were obtained, including at least one 16S rDNA and one *cox1* for each female and male of each tick species per district. Consensus sequences of ticks were obtained as follows: 16S rDNA for *Hy*. *turanicum* (410 bp), 16S rDNA for *Hy*. *asiaticum* (420 bp), *cox1* for *Hy*. *turanicum* (531 bp), and *cox1* for *Hy*. *asiaticum* (611 bp). Considering the closest identity, 16S rDNA and *cox1* sequences for *Hy*. *turanicum* were 100% identical with the corresponding species reported from Pakistan. Similarly, the 16S rDNA sequences for *Hy*. *asiaticum* showed a maximum identity of 99.52%, while its *cox1* sequences revealed a maximum identity of 100% with conspecific from China, Kazakhstan, and Turkey.

For each amplified fragment of *Rickettsia* spp., bidirectional sequences were obtained, which gave consensus sequences as follows: *gltA* (369 bp), *ompA* (471 bp), and *ompB* (790 bp) genes. The obtained sequences showed 100% identity with *R. aeschlimannii* reported from Egypt, Italy, Kazakhstan, Kenya, Pakistan, and Senegal.

Among the Anaplasmataceae, 8 of 80 (9.52%) samples were positive for 16S rDNA, including four *Hy. turanicum* and four *Hy. asiaticum* ticks. From the amplified fragments of Anaplasmataceae, bidirectional sequences were obtained, which resulted in the following consensus sequences: 16S rDNA (301 bp) for *Anaplasma* sp. and 16S rDNA sequence (333 bp) for *Ehrlichia* sp. The former sequence showed 99.67% identity with unknown *Anaplasma* sp. reported from Morocco, while the latter sequence revealed 100% identity with an undetermined *Ehrlichia* sp. reported from Pakistan.

The overall detection rates were as follows: 10% (8 of 80) for *R. aeschlimannii* associated with *Hy*. *turanicum*, 6.3% (5 of 80) for *Anaplasma* sp., and 6.3% (5 of 80) for *Ehrlichia* sp. associated with both *Hy*. *turanicum* and *Hy*. *asiaticum*. Additionally, coinfection with *R. aeschlimannii*, *Anaplasma* sp., and *Ehrlichia* sp. was found in 2 of 80 (2.5%) *Hy. turanicum* ticks. Detailed information about the infection rates of *R. aeschlimannii*, *Anaplasma* spp., and *Ehrlichia* spp. is shown in Table 2.

Following are the GenBank accession numbers of all consensus sequences of this study: 16S rDNA of *Hy*. *turanicum* (PQ218624) and *Hy*. *asiaticum* (PQ218629), *cox1* of *Hy*. *turanicum* (PQ218744), and *Hy*. *asiaticum* (PQ218788). *R. aeschlimannii*, *gltA* (PQ221477), *ompA* (PQ227817), and *ompB* (PQ227818), 16S rDNA for *Anaplasma* sp. (PQ213859), and *Ehrlichia* sp. (PQ214321).

### 3.4 Phylogenetic outcomes

**Figure 3:**
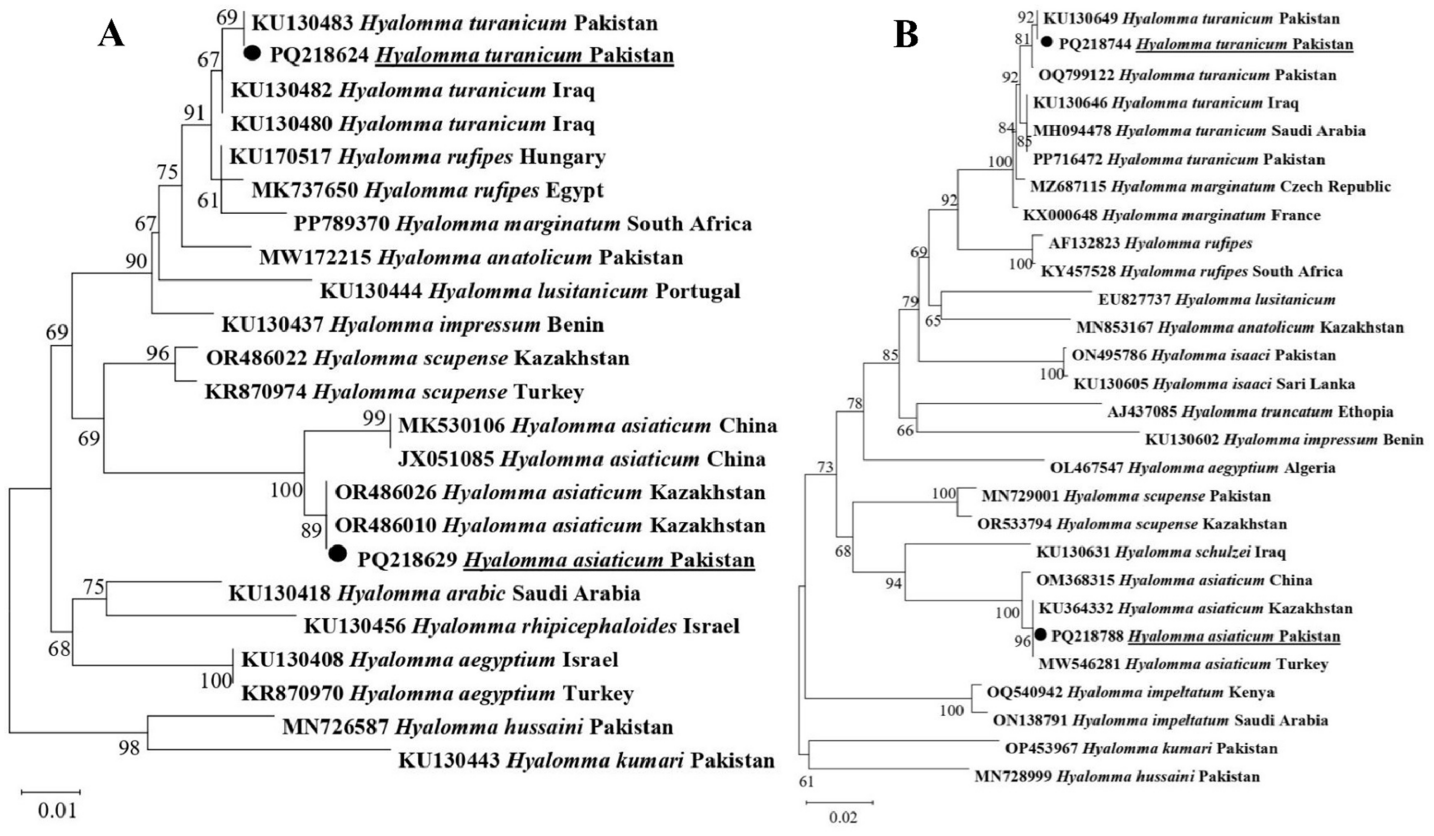
Phylogenetic tree constructed for *Hyalomma* spp. based on 16S rDNA **(A)** and *cox1* **(B)** sequences. Each sequence was identified by accession number, species name, and origin country. Sequences of *Hyalomma hussaini* and *Hyalomma kumari* were used as an outgroup. The acquired sequences have been highlighted.

The phylogenetic tree constructed 16S rDNA sequences revealed that *Hy*. *turanicum* clustered with the same species recorded from Iraq and Pakistan (KU130480, KU130482, and KU130483), while *Hy*. *asiaticum* grouped with the corresponding species reported from China and Kazakhstan (JX051085 MK530106, OR486026, and OR486010) (Figure 3A). The phylogenetic tree constructed for the *cox1* sequences showed that *Hy*. *turanicum* clustered with the related species from Saudi Arabia, Pakistan, and Iraq (OQ799122, PP716472, KU130649, KU130646, and MH094478), while *Hy*. *asiaticum* grouped with the related species described from Kazakhstan, China, and Turkey (OM368315, KU364332, and MW546281) (Figure 3B).

**Figure 4:**
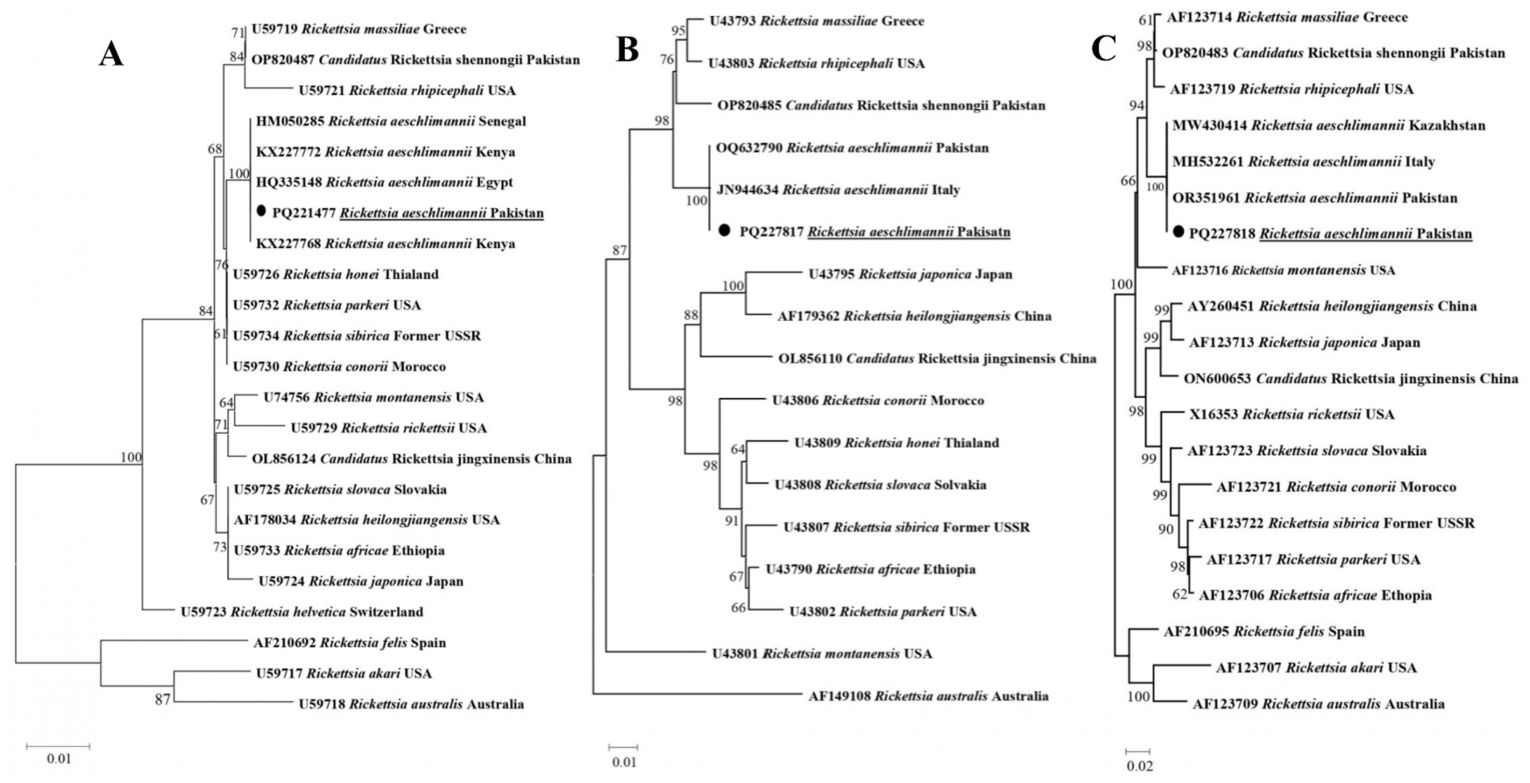
Phylogenetic tree constructed for *Rickettsia* spp. based on *gltA* **(A)**, *ompA* **(B)**, and *ompB* **(C)**. Each sequence was identified by accession number, species name, and origin country. *Rickettsia akari* and *Rickettsia australis* were jointly used as outgroups, and the acquired sequence has been highlighted.

In a phylogenetic tree developed for rickettsial *gltA*, *R. aeschlimannii* was detected in *Hy*. *turanicum* grouped with the sequences of the corresponding species described from Egypt, Kenya, and Senegal (HQ335148, KX227772, KX227768, and HM050285) (Figure 4A). In a phylogenetic developed for rickettsial *ompA*, *R. aeschlimannii* clustered with the sequences of the same species recorded from Italy and Pakistan (JN944634 and OQ632790) (Figure 4B). Additionally, in the phylogenetic tree obtained for rickettsial *ompB*, *R. aeschlimannii* grouped with the corresponding species reported from Italy, Kazakhstan, and Pakistan (MH532261, MW430414, and OR351961) (Figure 4C).

**Figure 5:**
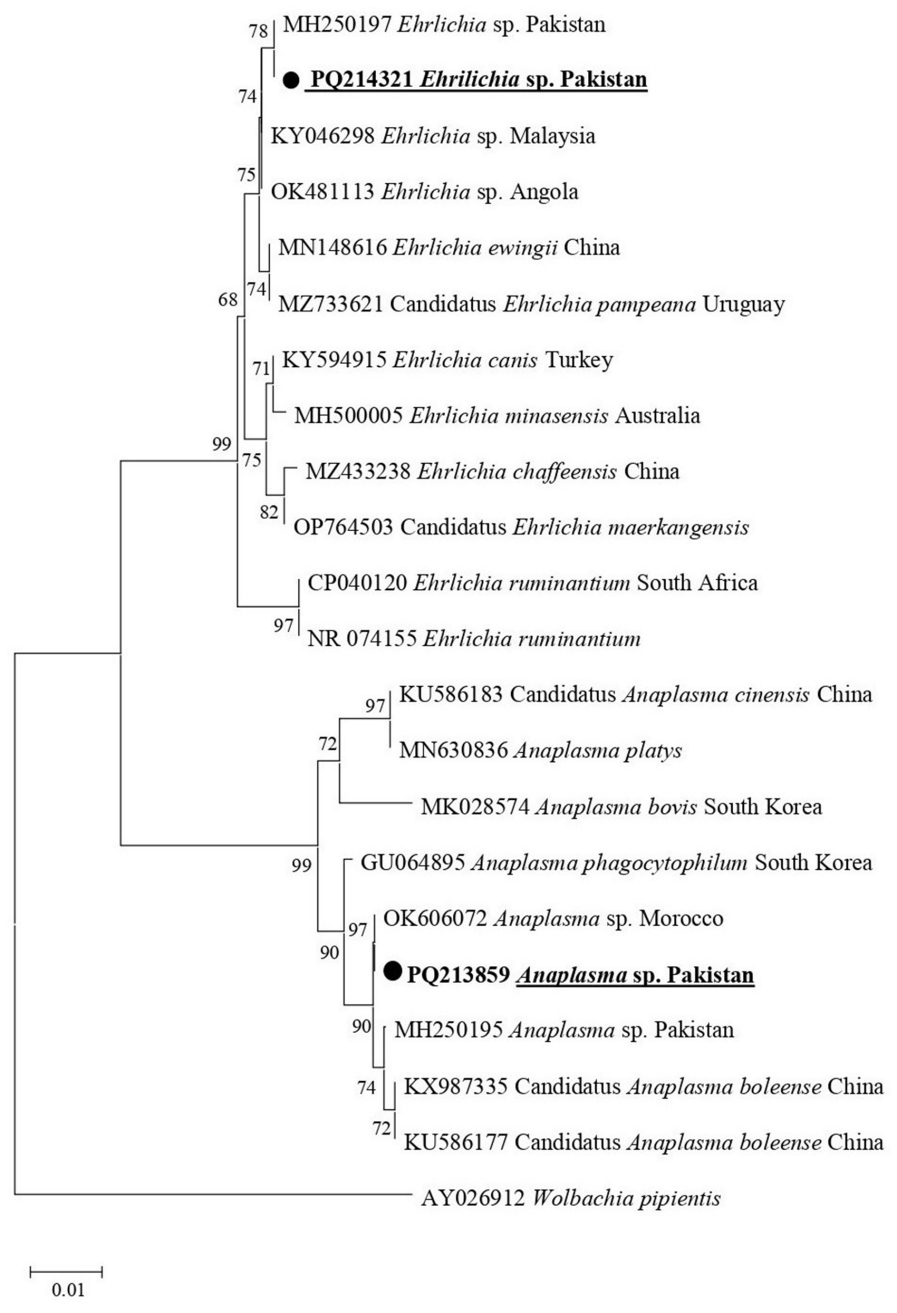
Phylogenetic tree constructed for 16S rDNA sequences of *Anaplasma* spp. and *Ehrlichia* spp. Each sequence was identified by providing the accession number, species name, and origin country. The sequence of *Wolbachia pipientis* was used as an outgroup, and the acquired sequences have been highlighted.

In the phylogenetic tree for Anaplasmataceae 16S rDNA sequences, *Anaplasma* sp. associated with *Hy*. *asiaticum* and *Hy*. *turanicum* was clustered with an undetermined *Anaplasma* sp. described from Morocco (OK606072) (Figure 5). In the same phylogenetic tree, *Ehrlichia* sp. recorded in the same ticks was grouped with an undermined *Ehrlichia* sp. previously reported from Pakistan (MH250197) (Figure 5).

## 4 Discussion

Sheep and goats are commonly raised animals in Pakistan, particularly in rural areas where these domestic animals serve as a significant source of income (34, 35). Despite the diverse range of ticks and TBDs, which cause significant economic losses in the livestock sector in rural areas of Pakistan, there is limited knowledge about the occurrence of *Hy*. *turanicum* and *Hy*. *asiaticum* ticks, as well as the pathogens associated with them. Few studies have been conducted to characterize *Hyalomma* ticks and/or associated pathogens from different regions of Pakistan (5–7, 9, 17). Herein, we morphologically and molecularly characterized two *Hyalomma* species, *Hy*. *turanicum* and *Hy*. *asiaticum,* for the first time from Balochistan, Pakistan. Additionally, this research also detected *R. aeschlimannii* in *Hy*. *turanicum* and undetermined *Anaplasma* sp. and *Ehrlichia* sp. in both *Hy*. *turanicum* and *Hy*. *asiaticum*.

Morphological identification of *Hyalomma* ticks, including *Hy*. *turanicum* and *Hy. asiaticum*, presents challenges (12, 15). In addition to morphological comparisons within their species, *Hy. turanicum* and *Hy. asiaticum* were also compared to their closest relatives within their respective complexes. *Hyalomma turanicum* was distinguished from *Hy. marginatum* by the presence of dense punctations on the conscutum or scutum, moderately narrow dorsal prolongation of the spiracular plate, and denser circumspiracular setae (12). Similarly, *Hy. asiaticum* was differentiated from *Hy. dromedarii* by characteristics such as: in males, a posteromedian groove that does not reach the parma and straighter adanal plates; and in females, a more U-shaped genital operculum and bulging preatrial fold (15).

To validate the morphological identification of ticks and ascertain the presence of a diverse array of pathogens, molecular confirmation holds significance (36, 37). Comprehensive molecular studies on the *Hyalomma* ticks have been conducted using the genetic markers; 16S rDNA and *coxI* (6, 7). In addition to clustering with the same species in the monophyletic clade, *Hy*. *turanicum* in the current study formed a sister clade with *Hy*. *marginatum* and *Hy*. *rufipus* within the subgenus *Euhyalomma* (38), corroborating previous studies that categorize them as members of the *Hy*. *marginatum* complex based on both morphological and genetic data (12, 38). Similarly, *Hy*. *asiaticum* clustered with the same species in the monophyletic clade and formed a sister clade with *Hyalomma schulzei* within the subgenus *Euhyalomma*, consistent with earlier studies that identify them as members of the *Hy*. *asiaticum* group, supported by morphological and genetic data (15, 38).

*Rickettsia* spp., including *R. aeschlimannii,* are zoonotic pathogens carried by different arthropod vectors, including *Hyalomma* ticks (18, 26, 39). The detection of *Rickettsia* spp. in ticks is significant not only for recognizing infected ticks but also for assessing the risk of transmission to humans (40). Considering suitability (41, 42), genetic markers such as *gltA*, *ompA*, and *ompB* were employed in this study to detect and phylogenetically analyze *Rickettsia* spp. in *Hyalomma* ticks. Previously, *R. aeschlimannii* has been detected in *Hy*. *turanicum*, *Ha*. *bispinosa*, and *Ha*. *montgomeryi* in Pakistan (43). This study further expanded the range of competent tick hosts and the geographical distribution of *R. aeschlimannii* in Pakistan by detecting it in *Hy*. *turanicum* in the Balochistan province. Transmission from animals to humans occurs through the bite of an infected tick, resulting in Mediterranean spotted fever (39). Additionally, *Rickettsia amblyommii*, *R. massiliae*, *Rickettsia conorii*, and *Rickettsia hoogstraalii* have been detected in other *Hyalomma* ticks, including *Hy*. *hussaini*, *Hy*. *anatolicum*, *Hy*. *dromedarii*, *Hy*. *Turanicum*, and *Hy*. *kumari* (7, 25, 44, 45). By clustering *R. aeschlimannii* with identical species from various regions within the spotted fever group, all three phylogenetic trees mutually validated each other. *Anaplasma* and *Ehrlichia* spp. are primarily transmitted by ticks and can cause diseases in humans and animals (46, 47). Partial fragments of 16S rDNA, widely acknowledged as a reliable molecular marker for characterizing tick-associated *Anaplasma* and *Ehrlichia* spp. (20), were utilized in this study. The detection of *Anaplasma* and *Ehrlichia* spp. in *Hyalomma* ticks in the present study, as well as *Anaplasma marginale*, *Anaplasma ovis*, and *Anaplasma centrale*, in other *Hyalomma* ticks, including *Hy*. *anatolicum* and *Hyalomma scupense,* and undetermined *Ehrlichia* spp. in *Hyalomma* ticks, including *Hy*. *dromedarii* and *Hy*. *anatolicum*, suggests a greater diversity of these tickborne microorganisms in Pakistan. Herein, *Anaplasma* sp. shared phylogenetic similarities with *Candidatus* Anaplasma boleense, and *Ehrlichia* sp. with *Ehrlichia ewingii*, indicating potential zoonotic implications. The detection of *R. aeschlimannii*, *Anaplasma* sp., and *Ehrlichia* sp. in *Hyalomma* ticks in the study region indicates risks to livestock workers and farmers, especially in rural areas. In addition, it may be associated with economic losses and food security by affecting livestock. Therefore, understanding various aspects of these bacteria, such as their ecology, genetic diversity, and pathogenicity, is necessary to mitigate the associated risks in Pakistan.

The presence of *Hy*. *turanicum* and *Hy. asiaticum*, along with their notable abundance in the Balochistan province of Pakistan, may be attributed to nomadic migration, resulting in the uncontrolled movement of domestic animals between Balochistan, Afghanistan, and Iran (48). Other factors contributing to the prevalence of these tick species could include the presence of suitable hosts and the arid or semi-arid climate of the study area (6, 26). Alternative approaches implemented in the study area, such as cohabiting with diverse hosts in shared habitats, managing densely populated herds, and employing mixed grazing methods, may positively impact the presence of these ticks. Additionally, these conditions may influence the association between *Hyalomma* ticks and bacterial species. As the present study is based on relatively a small size sample size and is restricted to the Balochistan province of Pakistan, future studies should aim to increase the sample size, expand the geographic coverage, and investigate the vector competence of *Hyalomma* species for associated bacteria.

## 5 Conclusion

This study addresses a significant knowledge gap concerning *Hy*. *turanicum* and *Hy*. *asiaticum*, which were morphologically and genetically characterized for the first time in Balochistan, Pakistan. Additionally, the study molecularly assessed *R. aeschlimannii* in *Hy*. *turanicum,* as well as *Anaplasma* sp. and *Ehrlichia* sp. in both *Hy*. *turanicum* and *Hy*. *asiaticum*. Notably, it records the coinfection of all these three bacterial species in *Hy*. *turanicum*. This study may help to understand the identity, molecular epidemiology, and geographic distribution of *Hyalomma* ticks and associated pathogens. The findings of the present study indicate that public awareness and surveillance of tick-borne diseases in the region are needed, which may mitigate the risks to public health and livestock.

## Data Availability

The datasets presented in this study can be found in online repositories. The names of the repository/repositories and accession number(s) can be found in the article/supplementary material.

## Footnotes

^1^ https://www.ncbi.nlm.nih.gov/
